# The Value of Belladonna as a Prophylactic in Scarlet Fever

**Published:** 1876-01

**Authors:** 


THE VALUE OF BELLADONNA AS A PROPHYLACTIC IN SCARLET FEVER. Edward B. Stephens, M.D., Professor of Materia Medica in Syracuse University, in a note to the Philadelphia *Medical Times* says: About the year 1860, perhaps,—I am not quite certain as to that—scarlet fever prevailed as an epidemic in Southern Ohio, especially so at Lebanon, where I then resided—whole families being swept away. At that time I experimented with belladonna very fully, and, as I think, completely, carefully, and honestly. I placed children of several families very fully under the influence of belladonna; I pushed the remedy until I secured the constitutional effects, especially the rash. When I got the eruption I stopped the administration of the belladonna; but I did in several families at that time push the belladonna until I had these well-marked constitutional manifestations. What was the result? Simply that scarlatina invaded those families just the same; and the most fatal cases I had in that epidemic were children who had been previously saturated to the degree of eruption by belladonna. I simply note this, as I think, very positive result of an experiment as to the prophylaxis of belladonna in scarlatina. My belief is that it has no control whatever.

